# Longitudinal trends of percutaneous endoscopic gastrostomy in geriatric hospital units in Germany

**DOI:** 10.1007/s41999-026-01425-x

**Published:** 2026-02-11

**Authors:** Peter Willschrei, Nina Rosa Neuendorff, Gero Lueg, Chantal Giehl, Maryam Pourhassan, Rainer Wirth

**Affiliations:** 1 Department of Geriatric Medicine, Kliniken Essen-Mitte, Evang. Krankenhaus Essen-Steele, Essen, Germany; 2 Department of Geriatric Medicine, Marien Hospital Herne, Ruhr-University Bochum, Hölkeskampring 40, 44625 Herne, Germany

**Keywords:** PEG, Gastrostomy, Tube feeding, Mortality, Older adults, Geriatrics

## Abstract

**Purpose:**

To quantify long-term trends in percutaneous endoscopic gastrostomy (PEG) use in geriatric inpatient care in Germany and describe associated diagnoses and in-hospital mortality.

**Methods:**

We conducted a nationwide retrospective cohort study using the GEMIDAS Pro registry (2006–2024) and included all patients aged ≥ 60 years. According to the database items, we received information about the incidence of PEG placement, nutrition-relevant diagnoses, patients age, functional and mental status, length of hospital stay, where patients were admitted from and discharged to and the hospital mortality of patients with and without PEG placement.

**Results:**

Among 1,355,436 admissions (≥ 60 y; mean age 83y; 67% women), 4091 (0.3%) underwent PEG placement. Annual prevalence declined from 1.31% (2006) to 0.07% (2024, *p* < 0.001), a 94.7% relative reduction (absolute decrease 1.24 percentage points). Binary logistic regression confirmed a 10% per-year reduction in the odds of PEG placement (OR 0.90, 95% CI 0.894–0.905; *p* < 0.001). Among PEG recipients, stroke was the most frequent nutrition-relevant main diagnosis and dysphagia the most frequent secondary diagnosis. Dementia was the main indication in only 0.8% of PEG recipients, and as a secondary diagnosis it declined from 40% (2006) to 15% (2024, *p* = 0.021). In-hospital mortality was higher with PEG than without (11% vs 3%, *p* < 0.001); mortality among PEG recipients decreased with an undulating course over time (24.3% in 2006 to 7.4% in 2024, *p* < 0.001).

**Conclusion:**

PEG use in German geriatric units declined substantially during the last two decades. The pattern aligns with a more selective, guideline-concordant practice focused on neurogenic dysphagia, alongside reduced use in dementia.

## Key summary points

**Aim:**

Track how often percutaneous endoscopic gastrostomy (PEG) tubes are used in German geriatric inpatient care (2006–2024), associated nutritionally relevant diagnoses and in-hospital mortality.

**Findings:**

Among 1,355,436 admissions (≥ 60 y; mean 83 y; 67% women), 0.3% had a PEG insertion during hospital stay. Incidence fell sharply from 1.31% (2006) to 0.07% (2024). Stroke was the most common main diagnosis; dysphagia the most common secondary diagnosis among PEG recipients. In-hospital mortality was 11% with PEG vs 3% without, and mortality in PEG recipients decreased slightly over time.

**Message:**

PEG use in German geriatric units fell markedly, most likely due to more restrictive patient selection and evolving practice.

## Introduction

Percutaneous endoscopic gastrostomy (PEG) provides gastral access for patients in need of long-term tube feeding or in patients with an indication for short-term tube feeding who do not want or tolerate a nasogastric tube [1, 2]. Since its first description by Gauderer and Ponsky in 1980 [3–5], PEG tube placement has become a widely used and generally safe technique across different indications and all age groups.

In general, tube feeding is indicated if oral nutrition is predicted to be impossible for more than 3 days or if oral nutrition covers less than 50% of the nutritional demands for more than one week [2]. Enteral access can be achieved via nasogastric tubes (NGT) for short-term feeding or PEG, if tube feeding is expected to be necessary for more than 4 weeks [2].

Consequently, prevalence varies by population and health-system practice. In paediatrics, prevalence of PEG insertion is very low but higher in those with major congenital anomalies, with higher rates in Nordic countries and the UK than in Italy/Spain [6]. Among adults, especially geriatric patients, PEG use is more common yet heterogeneous; for example, in a German cohort of acute care geriatric patients from 2004, a 1.4% incidence of PEG tube placement during hospital stay was reported [7]. Patterns in home enteral nutrition (HEN) also differ: HEN use is increasing overall [8], Poland relies mainly on gastrostomy (~ 77% PEG tube placement among HEN patients) [9], whereas Italy more often uses NGT (e.g., 60% in neurological disease, 36% in head-and-neck cancer, 23% abdominal cancer patients on NGT) [8], and Spain’s registry reports PEG tube placement in only 6.8–10% of HEN patients [10]. These disparities reflect differences in underlying diagnoses (neurologic disease, cancer, congenital anomalies) and in national care pathways and access models.

Whether PEG tube placement results in a net benefit for the patients depends on patient selection and goals of care. However, as there are no prospective randomized controlled trials, the indication for a PEG may be rather subjective and dependent on physician’s expertise and experience. From a medical perspective, the indication is often a case-by-case decision. Clinical experience and available data suggest that patients with severely impaired general and nutritional status derive limited benefit from PEG feeding, whereas PEG tube placement can be valuable when the cause of malnutrition, e.g. dysphagia, is reversible or expected to improve (e.g., after an acute stroke) [11]. On the other hand, findings of a cohort study among 36,492 nursing home residents with advanced dementia, demonstrated that neither feeding via PEG feeding nor the timing of the insertion improved survival [12]. Accordingly, major guidelines urge caution in situations unlikely to improve outcomes, most notably advanced dementia, and call for better risk stratification to identify appropriate candidates [13–16].

In geriatrics, the risk–benefit balance remains uncertain because prospective randomized trials are lacking, and complication rates are not negligible [17]. A large retrospective analysis of the U.S. Nationwide Inpatient Sample (2006) reported 10.8% in-hospital mortality after PEG tube placement, underscoring the need for careful candidate selection and attention to complications, quality of life, and costs [18]. Echoing this, twenty years after the introduction of PEG feeding, Gauderer emphasized that technical progress alone is insufficient; clinicians must carefully evaluate prognosis, balance benefits and burdens, and align decisions with the patient’s preferences and values, ensuring that PEG tube placement is pursued only when it is likely to provide meaningful benefit [19].

In Germany, PEG placement is mainly performed during a hospital stay due to an acute disease or deterioration of chronic disease. As for any other invasive medical procedure, a written informed consent and an expected benefit for the patient is necessary to perform the procedure. In principle, the procedure may be applicable for a patient of any medical department. In the past however, a majority of PEG placements have been performed in geriatric patients. A prior Geriatric Minimum Data Set (GEMIDAS) analysis from 2004 reported a 1.4% incidence of PEG tube placement among hospitalized patients in geriatric wards in Germany [7] and in 2007, 5.6% of all nursing home residents in Germany were reported to be fed via a PEG [20]. Since then, subsequent guideline updates, accumulating clinical experience, and evolving nutritional medicine have likely influenced PEG use in geriatric medicine. Experts have the impression that the number of PEG insertions decreased substantially but this has not yet been studied.

Accordingly, the primary objective of this study was to examine temporal trends in the incidence of PEG tube placement in German geriatric acute care units using an updated version of the same registry (GEMIDAS Pro) for 2006–2024. Secondary objectives were to compare patient outcomes by PEG status and to characterize features associated with PEG tube placement use.

## Subjects and methods

### Data source (GEMIDAS Pro registry)

The Geriatric Minimum Data Set (GEMIDAS) is a nationwide, voluntary quality-assurance registry for inpatient geriatrics coordinated by the Bundesverband Geriatrie (BVG) in Germany. Many geriatric hospital departments in Germany participate in this project and provide anonymized data about the individual hospital stay of each patient. The GEMIDAS database captures treatment-related information such as ICD diagnosis codes, procedure codes, length of stay, results of the standardized geriatric assessment at admission and at discharge, and some additional variables. In particular, we asked to search for the OPS code 5-431.2 which identifies persons who received a PEG tube during hospital stay. After permission by the board of the registry, we received the anonymized data of all acute care patients in August 2024. Because the registry’s hosting and management changed over time and the database was upgraded to GEMIDAS Pro in 2006, we had access only to GEMIDAS Pro data, which begin in autumn 2006.

### Study design and participants

We conducted a retrospective observational cohort analysis of routinely collected inpatient data from the GEMIDAS Pro registry covering 2006–2024 (18 years), with incomplete data in 2006 and 2024. A prior analysis from 2004 using an earlier version of the GEMIDAS registry [7] was included only to anchor the time-trend figure but was not re-analysed in this study; no data were available for 2005 due to the registry transition/adaptation between GEMIDAS versions in that year. The analytic dataset included acute geriatric departments nationwide with complete procedure-code capture and transmission, comprising 1,403,790 inpatient admissions, of which 4251 involved PEG tube placement.

### Measurements

Extracted variables were age, sex, length of stay, in-hospital mortality, admission source, and discharge destination. Functional status was assessed using the Barthel Index (BI) to quantify self-care performance (at admission and, where available, at discharge) [21], and cognitive status using the Mini-Mental State Examination (MMSE) at admission [22, 23]. Diagnosis profiling included nutrition-relevant ICD-10 codes (both main and secondary). Procedures were identified via DRG/OPS codes, including PEG tube placement (OPS 5-431.2).

### Diagnosis definitions

For the purpose of this analysis, only nutritionally relevant diagnoses were extracted from the database. Main and secondary diagnoses were coded according to ICD-10-GM (2024) and analysed using a prespecified, nutrition-relevant panel comprising dementia (F00–F03, G30), stroke (I61–I64), depression (F30–F39), Parkinson’s syndrome (G20–G22), malignant neoplasms of the mouth and pharynx (C00–C14), malignant neoplasms of the digestive tract (C15–C26), aspiration pneumonia (J69.0), delirium (F05), dysphagia (R13), dehydration (E86), unintentional weight loss (R63.4), cachexia (R64), anorexia (R63.0), and malnutrition (E40–E46). In the German DRG-system the main diagnosis is defined as the condition that, was primarily responsible for the hospital admission. Secondary diagnoses were conditions coexisting at admission or arising during the stay that required evaluation, treatment, monitoring, and/or nursing care.

### Data analysis

The statistical analysis was conducted using SPSS Statistics for Windows software (Version 29.0, IBM Corp, Armonk, NY, USA). Continuous variables were represented by means and standard deviations (SDs) for normally distributed data, and by medians with interquartile ranges (IQR) for data not adhering to a normal distribution. Absolute numbers and relative frequencies (%) were used to express categorical variables. Between-group differences (PEG tube placement vs. no PEG tube placement and mortality vs. no mortality) were assessed using unpaired t-tests for normally distributed continuous variables, Mann–Whitney U tests for non-normally distributed continuous/ordinal variables, and Pearson’s *χ*^2^ test for categorical variables.

For each diagnosis, the prevalence within PEG and within no-PEG tube placement groups separately, for main and secondary diagnoses was calculated. Differences were expressed as absolute percentage-point contrasts (Δ = %PEG − %no-PEG) and, diagnoses were ordered by |Δ| within the main and secondary blocks. Annual PEG incidence was calculated as the percentage of all admissions per calendar year with PEG tube placement. Year-to-year differences in annual PEG tube placement incidence and in the proportion of PEG recipients with main diagnoses and selected secondary diagnoses were assessed using Pearson’s *χ*^2^ across years. To quantify the temporal trend in PEG use, we fitted a binary logistic regression analysis with PEG placement (yes/no) as the dependent variable and calendar year (centered at 2006) as a continuous independent variable; effects are reported as odds ratios (OR) per year with 95% confidence intervals. For interpretability, we also expressed multi-year effects over 5, 10, 15, and 18 years as ${\mathrm{OR}}_{k{\mathrm{y}}}=({\mathrm{OR}}_{\text{per year}}{)}^{k}$(e.g., $k=\mathrm{5,10,15,18}$).

To identify factors independently associated with PEG placement, we fitted a second binary logistic model (dependent variable: PEG yes/no) including age (years), sex (male/female), Barthel Index at admission (per point), MMSE (per point), and nutrition-relevant main diagnoses entered as independent indicators. Results are presented as adjusted ORs with 95% CIs. Moreover, to test the relationship between PEG and adverse outcome, we fitted a third binary logistic regression analysis for in-hospital mortality (dependent variable: died yes/no) with PEG status and the same covariates as above (age, sex, Barthel, MMSE, and the diagnosis indicators).

To examine whether a greater number of secondary diagnoses was associated with PEG tube placement use, we counted the total number of secondary diagnoses per admission and compared PEG vs. no-PEG tube placement across this count. In-hospital mortality was compared between PEG and no-PEG tube placement groups using Pearson’s *χ*^2^. A p-value of less than 0.05 was considered statistically significant.

### Ethical approval

This study complies with the Declaration of Helsinki. The study was approved by the ethical committee of Ruhr-University Bochum with the comment that a formal approval is not mandatory for the analysis of entirely anonymized data (Reg.Nr: 2025-068-f-N).

## Results

### Baseline characteristics

Baseline characteristics of the study cohort are summarized in Table 1. The source database contained 1,403,790 hospital admissions. Of these, 4980 (0.4%) were patients younger than 60 years. As our focus was geriatric patients, we excluded all patients < 60 years from subsequent analyses. The final analytic cohort therefore comprised 1,355,436 patients aged ≥ 60 years. In this cohort, the mean age was 82.6 ± 6.9 years and 67% were women. Most patients had no PEG tube placement (*n* = 1,351,345; 99.7%), while 4091 (0.3%) had a PEG tube placement during their hospital stay.

**Table 1:** Characteristic of study population

	Patients without PEG tube placement (*n* = 1.351.345, 99.7%)	Patients with PEG tube placement (*n* = 4091, 0.3%)	*P* value
Gender (*n*, %)			
Female	899.167 (67)	2159 (54)	< 0.001
Age (y), mean ± SD	82.6 ± 6.9	81.4 ± 6.9	< 0.001
MMSE, median (IQR)	25 (20–28)	18 (10–24)	< 0.001
BI on admission, median (IQR)	40 (25–55)	5 (0–15)	< 0.001
BI at discharge, median (IQR)	65 (40–80)	10 (5–15)	< 0.001
Length of hospital stay, median (IQR)	18 (15–22)	23 (19–30)	< 0.001
Admission from (*n*; %)			
Private household	274.466 (20.3)	602 (14.7)	< 0.001
Nursing facility	37.207 (2.8)	295 (7.2)	
Rehabilitation facility	4.024 (0.3)	11 (0.3)	
Short-term care	11.862 (0.9)	18 (0.4)	
Hospital (internal transfer)	335.411 (24.8)	1.038 (25.4)	
Hospital (external transfer)	581.958 (43.0)	1.811 (44.3)	
Other professional institution	5.508 (0.4)	18 (0.4)	
Data unavailable	100,909 (7.5%)	298 (7.3%)	
Discharge to (*n*; %)			
Private household	860.831 (63.7)	1.037 (25.3)	< 0.001
Nursing facility	162.429 (12.0)	1.363 (33.3)	
Rehabilitation facility	32.945 (2.4)	374 (9.1)	
Short-term care	103.861 (7.7)	497 (12.1)	
Hospice care	2.070 (0.2)	16 (0.4)	
Hospital (internal transfer)	20.997 (1.6)	61 (1.5)	
Hospital (external transfer)	54.389 (4.0)	93 (2.3)	
Hospital mortality	39.966 (3.0)	443 (10.8)	
Other professional institution	18.630 (1.4)	90 (2.2)	
Data unavailable	55,227 (4.1%)	117 (2.9%)	
Hospital mortality (*n*, %)			
Yes	39.966 (3.0)	443 (10.8)	< 0.001

*PEG* percutaneous endoscopic gastrostomy, *MMSE* mini mental state examination, *BI* Barthel-Index, Values are given as number (%), mean ± SD or median (*IQR* interquartile range)

Compared with patients without PEG tube placement, patients with PEG insertion were on average younger and more often male, had significantly poorer cognitive and functional status at admission and discharge, longer hospital stay, and less frequent discharge to home. They were less often admitted from private households and more frequently from nursing facilities and were more often discharged to nursing facilities (all *p* < 0.001). In-hospital mortality was higher among patients with PEG tube placement (10.8% vs. 3.0%; *p* < 0.001).

### Longitudinal trend in PEG tube placement

**Fig. 1:**
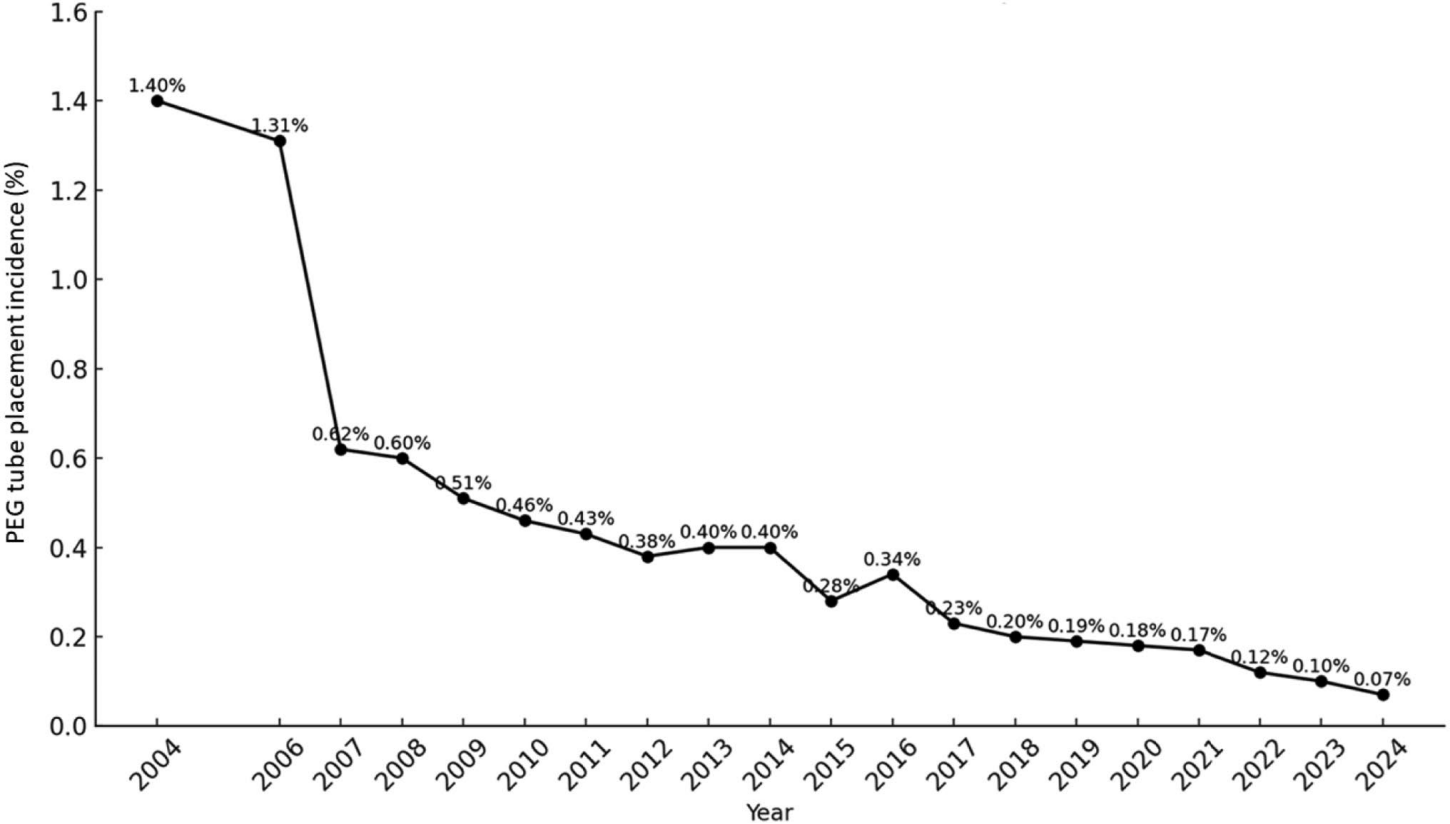
PEG tube placement incidence by year (2004, 2006–2024); *PEG* percutaneous endoscopic gastrostomy

The incidence of PEG placement by year is shown in Fig. 1. In 2004, incidence was 1.40% (previously published and included here for continuity [7]); no data were available for 2005. From 2006 onward, incidence declined from 1.31% to 0.07% in 2024 (−1.24 percentage points; −94.7% relative), with a marked step between 2006 and 2007 (from 1.31 to 0.62%) and a constant decline from then on. A binary logistic regression (2006–2024) confirmed that each additional calendar year was associated with 10% lower odds of PEG placement (*B* = −0.106; OR per year = 0.90; 95% CI 0.894–0.905; *p* < 0.001), equivalent to roughly 41% lower odds over 5 years, 65% over 10 years, 80% over 15 years, and 85% over 18 years.

### Incidence of PEG tube placement by diagnosis

**Fig. 2:**
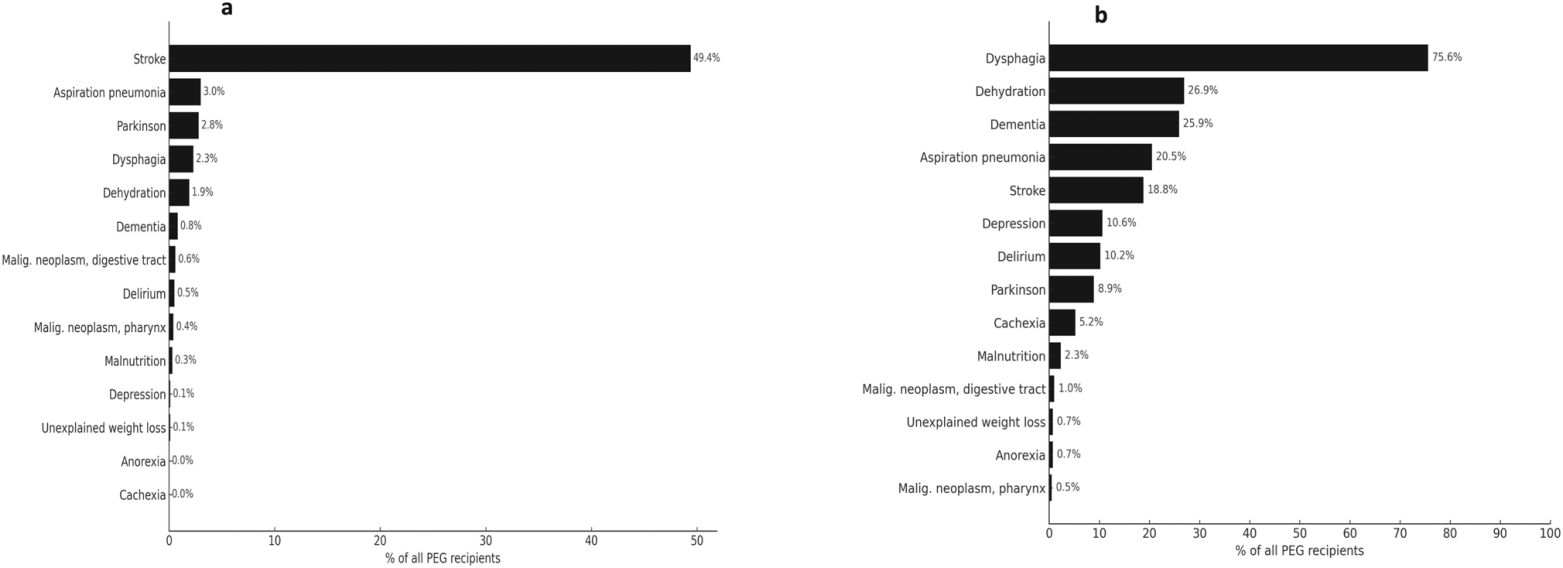
Distribution of main (**a**) and secondary (**b**) nutrition-relevant diagnosis among PEG tube placement recipients; PEG, Percutaneous endoscopic gastrostomy

Incidence of PEG tube placement by main and secondary diagnoses is summarized in Table 2 (diagnoses ordered by absolute between-group differences) and Fig. 2a and b.

**Table 2:** Prevalence of PEG tube use by diagnosis

Diagnosis	Patients without PEG tube (*n*, %)	Patients with PEG tube (*n*, %)	Δ% (PEG – No PEG)	*P* value
Main diagnosis				
Stroke (I61–I64)	107,357 (7.9)	2020 (49.4)	+ 41.5	< 0.001
Aspiration pneumonia (J69.0)	3284 (0.2)	121 (3.0)	+ 2.8	< 0.001
Dysphagia (R13)	829 (0.1)	96 (2.3)	+ 2.2	< 0.001
Parkinson (G20–G22)	12,957 (1.0)	115 (2.8)	+ 1.8	< 0.001
Dehydration (E86)	23,637 (1.7)	78 (1.9)	+ 0.2	0.440
Malnutrition (E40–E46)	569 (0.0)	11 (0.3)	+ 0.3	< 0.001
Dementia (F00–F03)	6891 (0.5)	31 (0.8)	+ 0.3	0.031
Unexplained weight loss (R63.4)	1055 (0.1)	4 (0.1)	0.0	0.568
Cachexia (R64)	183 (0.0)	1 (0.0)	0.0	0.427
Anorexia (R63.0)	106 (0.0)	2 (0.0)	0.0	0.043
Malignant neoplasm, pharynx (C00–C14)	347 (0.0)	16 (0.4)	+ 0.4	0.015
Delirium (F05)	10,400 (0.8)	22 (0.5)	−0.3	0.111
Depression (F30–F39)	4201 (0.3)	4 (0.1)	−0.2	0.010
Malignant neoplasm, digestive tract (C15–C26)	19,856 (1.5)	26 (0.6)	−0.9	< 0.001
Secondary diagnosis				
Dysphagia (R13)	82,586 (6.1)	3,093 (75.6)	+ 69.5	< 0.001
Aspiration pneumonia (J69.0)	9586 (0.7)	838 (20.5)	+ 19.8	< 0.001
Stroke (I61–I64)	40,279 (3.0)	769 (18.8)	+ 15.8	< 0.001
Dehydration (E86)	156,744 (11.6)	1102 (26.9)	+ 15.3	< 0.001
Dementia (F00–F03)	208,032 (15.4)	1060 (25.9)	+ 10.5	< 0.001
Parkinson(G20–G22)	57,125 (4.2)	364 (8.9)	+ 4.7	< 0.001
Delirium (F05)	90,806 (6.7)	416 (10.2)	+ 3.5	< 0.001
Cachexia (R64)	29,748 (2.2)	214 (5.2)	+ 3.0	< 0.001
Anorexia (R63.0)	3636 (0.3)	29 (0.7)	+ 0.4	< 0.001
Malignant neoplasm, pharynx (C00–C14)	955 (0.1)	21 (0.5)	+ 0.4	< 0.001
Unexplained weight loss (R63.4)	8728 (0.6)	29 (0.7)	+ 0.1	0.568
Malig. neoplasm, digestive tract (C15–C26)	12,945 (1.0)	42 (1.0)	+ 0.1	0.638
Malnutrition (E40–E46)	34,710 (2.6)	96 (2.3)	−0.3	0.399
Depression (F30–F39)	155,857 (11.5)	434 (10.6)	−0.9	0.067

*PEG* percutaneous endoscopic gastrostomy; The percentages are within PEG tube placement status

#### Main diagnosis

Among patients with PEG tube placement, stroke was the most frequent main diagnosis (49.4%), followed by aspiration pneumonia, Parkinson’s disease, and dysphagia (Fig. 2a). Associations with PEG insertion were modest for malnutrition and dementia, with no significant difference for dehydration, unexplained weight loss, or cachexia. PEG tube placement use was less frequent in patients with malignant neoplasms of the digestive tract and in those with depression. Over the study period, the proportion of PEG recipients with stroke as the main diagnosis declined substantially from 61.4% in 2006 to 37.0% in 2024 (*p* < 0.001), whereas aspiration pneumonia (*p* = 0.065), Parkinson’s disease (*p* = 0.119), and dysphagia (*p* = 0.190) showed no significant temporal change.

#### Secondary diagnosis

Among patients with PEG tube placement, dysphagia was by far the most common secondary diagnosis (75.6%), followed by dehydration, dementia, aspiration pneumonia, and stroke (Table 2, Fig. 2b). In contrast, malnutrition showed no meaningful difference, and unexplained weight loss and digestive-tract malignancy were similar between groups; depression was slightly less common among PEG recipients. Across study years, the proportion of PEG recipients with dysphagia remained consistently high, increasing from 64.3% in 2006 to 81.5% in 2024 (*p* < 0.001 for trend). In contrast, the proportion with dementia declined substantially from 40% in 2006 to 15.0% in 2024 (*p* = 0.021).

In binary logistic regression, dysphagia was the strongest independent predictor of PEG placement (OR 53.60, 95% CI 36.09–79.60; *p* < 0.001), followed by aspiration pneumonia (OR 14.37, 10.39–19.88; *p* < 0.001), malnutrition (OR 14.18, 5.18–38.81; *p* < 0.001), stroke (OR 5.77, 5.10–6.53; *p* < 0.001), Parkinson’s disease (OR 3.45, 2.50–4.76; *p* < 0.001), and dementia (OR 2.68, 1.51–4.78; *p* = 0.001). Male sex was also associated with higher odds (OR 1.67, 1.48–1.87; *p* < 0.001).

### Mortality

**Fig. 3:**
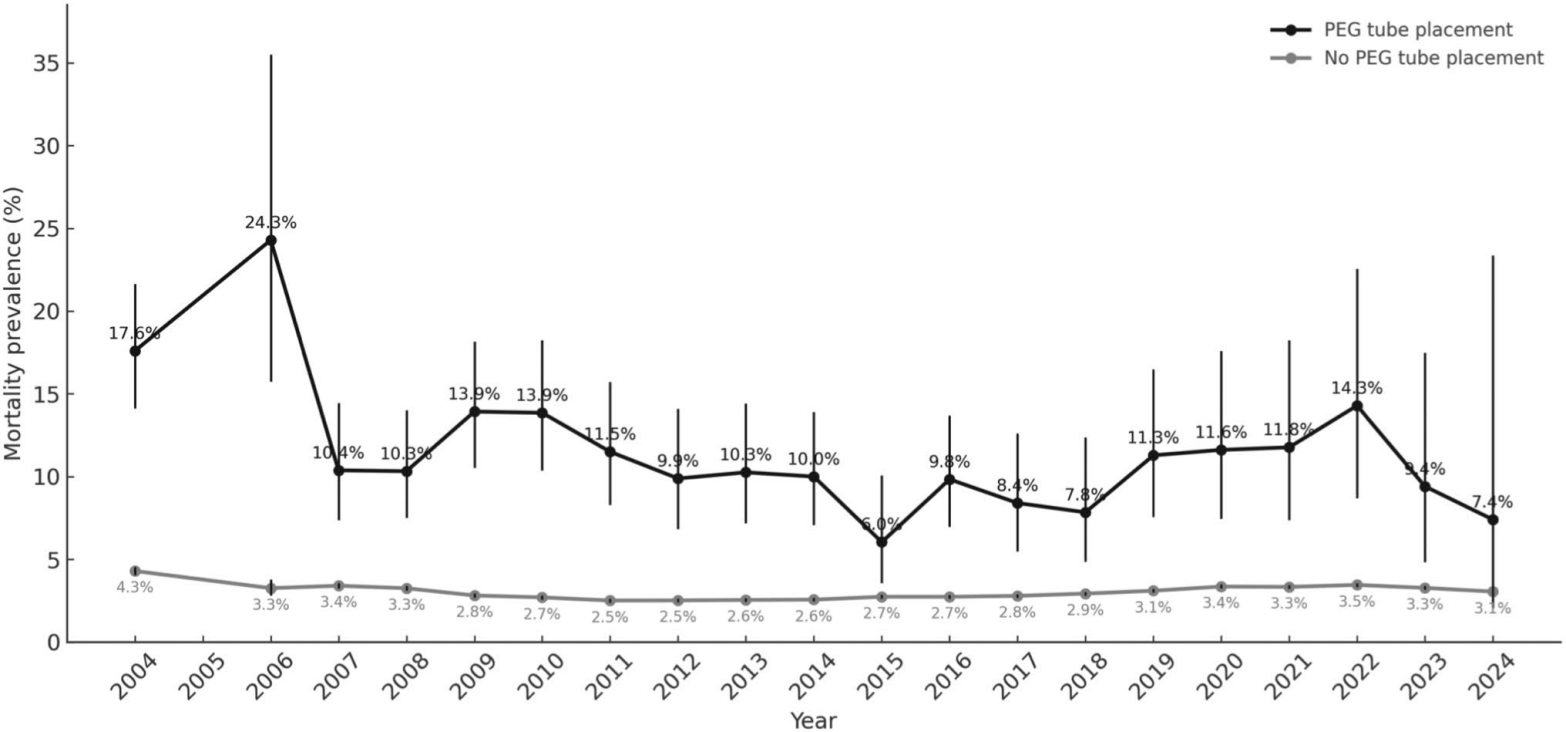
Annual in-hospital mortality prevalence (%) among patients with PEG (black line) and without PEG (gray line) tube placement in German acute geriatric units, 2006–2024. Points show observed prevalence; vertical bars indicate 95% confidence intervals (CIs). Sample sizes vary markedly by year (PEG tube placement: ~ 27–339; No-PEG tube placement: ~ 5000–102,000 per year), which explains the wider CIs for the PEG tube placement group and the barely visible (narrow) CIs for the No-PEG tube placement group; PEG, Percutaneous endoscopic gastrostomy

As shown in Fig. 3, in 2004 the in-hospital mortality prevalence after PEG tube placement was 17.5% (previously published and included here for continuity [7]; no 2005 data due to registry transition), compared with 4.3% in patients without PEG. Thereafter, mortality prevalence in the PEG tube placement declined with fluctuations from 24.3% (2006) to 7.4% (2024). In the No-PEG tube placement group, mortality remained low and stable (roughly 2.5–3.5%) with a slight gradual increase over time. Across all years, mortality was consistently and significantly higher in PEG tube placement recipients than in non-recipients, although CIs were wider for the PEG group in years with small *n*.

Within PEG recipients (Table 3), deaths were associated with older age, male sex, and poorer cognition at admission, and a shorter length of stay—consistent with death truncating hospital stay—while Barthel Index at admission, admission source and overall secondary-diagnosis count did not differ meaningfully.

**Table 3:** Differences between patients with and without in-hospital mortality among PEG recipients

	No mortality (*n* = 3648, 89.0%)	Mortality (*n* = 443, 11.0%)	*P* value
Gender (*n*, %)			
Female	1947 (54)	212 (49)	0.026
Age (y), mean ± SD	81.3 ± 6.9	82.2 ± 6.9	< 0.001
MMSE, median (IQR)	18 (10–24)	14 (8–20)	< 0.001
BI on admission, median (IQR)	5 (0–15)	5 (0–15)	0.112
Admission from (*n*; %)			
Private household	532 (15)	70 (16)	0.922
Nursing facility	263 (7)	32 (7)	
Rehabilitation facility	11 (0)	0 (0)	
Other professional institution	15 (0)	3 (1)	
Short-term care	17 (1)	1 (0)	
Hospital (internal transfer)	925 (26)	113 (26)	
Hospital (external transfer)	1619 (45)	192 (44)	
Data unavailable	214 (6)	29 (7)	
Length of hospital stay, median (IQR)	24 (19–30)	20 (13–27)	< 0.001
Total number of secondary diagnoses	2 (1–3)	2 (1–3)	0.355

*PEG* percutaneous endoscopic gastrostomy, *MMSE* mini mental state examination, *BI* Barthel-Index; Values are given as number (%), mean ± SD or median (IQR, interquartile range)

In binary logistic regression using mortality as dependent variable, PEG placement was associated with lower odds of in-hospital death (OR 0.40, 95% CI 0.33–0.49; *p* < 0.001). Mortality risk increased with age (OR 1.04 per year; *p* < 0.001) and was higher in males (OR 1.78; *p* < 0.001). Better function and cognition were protective (Barthel at admission OR 0.97 per point; MMSE OR 0.96 per point; both *p* < 0.001). Among main diagnoses, malnutrition (OR 2.13, 1.21–3.75), dysphagia (OR 1.90, 1.23–2.89), and aspiration pneumonia (OR 2.56, 2.09–3.13) were linked to higher mortality, whereas the adjusted odds were lower for stroke (OR 0.67), Parkinson’s disease (OR 0.41), dementia (OR 0.33) and depression (OR 0.27; all *p* ≤ 0.001 except depression p = 0.001).

### Number of secondary diagnoses and PEG tube placement use

Among patients with PEG tube placement, the median number of nutritionally relevant secondary diagnoses was 2 (IQR 1–3; mean 2.08). Most PEG tube placement recipients had 1–3 secondary diagnoses (1: 25.5%, 2: 33.3%, 3: 22.1%); few had none (7.5%) or ≥ 4 (11.6%). In the full cohort, the probability of receiving a PEG tube placement increased with diagnosis count, ~ 0.04% with 0 diagnoses, ~ 0.9% with 2, ~ 1.8% with 3, and ~ 3–5% with 4–6; the 7-diagnosis category appeared higher but was very rare.

## Discussion

Adequate indications for PEG tube placement remain a controversial issue in medicine with a variety of indications for its placement [24, 25]. Particularly in geriatric populations, the mortality after PEG insertion is high [26] and complications appear not negligible [17], hence the net benefit from PEG remains unclear [27]. In the present study, the nationwide analysis of GEMIDAS Pro data provides a comprehensive overview of PEG tube placement use in German geriatric inpatient care. Across 18 years and 1,355,436 admissions (≥ 60 y), 0.3% underwent PEG tube placement. Utilization declined markedly, from 1.31% in 2006 to 0.07% in 2024 (*p* < 0.001), an absolute decrease of 1.24 percentage points and 94.7% relative reduction (2006–2024). Binary logistic regression confirmed a 10% per-year reduction in the odds of PEG placement (OR 0.90, 95% CI 0.894–0.905; *p* < 0.001). Stroke and dysphagia were the most frequent nutritionally relevant main and secondary diagnosis among PEG recipients, respectively. Overall, in-hospital mortality was 11% among PEG recipients compared with 3% in patients without PEG. Notably, the marked decline of PEG tube placement use is not confined to geriatric units. Publicly available outputs from DESTATIS (Federal Statistical Office) with general hospital statistics including procedure-specific prevalence (e.g. PEG), indicate a similar downward trend in PEG use both in the general and older population across the German hospital system over time [28]. While interpreting the data of this study, it must be noted, that due to the large sample size, even small differences may be significant. Therefore, the magnitude of differences should preferably be considered. In addition, associations cannot prove causality and our interpretation of results are therefore sometimes obvious but mainly hypothetical.

Since its introduction in 1980 [3–5], PEG rapidly became a widely performed endoscopic procedure, with sustained growth documented across multiple health systems [29, 30]. In recent years, however, several health systems have reached a plateau or shown a subsequent decline in PEG utilization—consistent with our findings. For example, a Japanese national survey and systematic review reported a peak in PEG utilization in 2007 and a marked decline after 2015, coinciding with the 2014 fee-schedule revision and increasing acknowledgment of limited benefit in frail older adults [31]. Complementing this, Hattori et al. [32] observed a decline in gastrostomies among older adults from 2014 to 2016, followed by relative stability; age-stratified analyses showed a 33% reduction among those ≥ 90 years and a 6.1% decline in the 65–69 group between 2014–2019 [32]. Stated reasons for reduced PEG use in aforementioned studies include changes in the health insurance system, reimbursement-related factors, and emotional and ethical barriers [31, 32]. Furthermore, temporary system-wide downturns were also noted during the COVID-19 period; In a nationwide analysis from Poland [9], although PEG insertions increased annually from 2010–2019 but fell by 12.4% in 2020 in comparison with 2019, driven largely by a 40% reduction in cancer patients, due the limitations in medical procedures during the COVID-19 pandemic, also reported in the studies conducted in the the UK [33] and USA [34].

In general, there has been an increase in PEG insertions during the first 2–3 decades after the introduction of the endoscopic method in 1980. In the United States, an estimated 100,000–125,000 PEGs are placed annually [35–37], and 1993–2003 Nationwide Inpatient Sample (NIS) data show a 38% increase among adults ≥ 65 years, including greater use in Alzheimer’s dementia rising from ~ 5% to ~ 10% over the same period [29]. National reports show similar expansion elsewhere. For example, in Taiwan, incidence increased from 0.1 to 3.3 per 10^5^ inhabitants overall between 1997 and 2010, with a more pronounced rise among older persons (0.9 to 19.0 per 10^5^) [38]. However, in some nations the constant increase of PEG has been reported even during the 4th decade after the introduction of the endoscopic procedure. A Swedish registry study (1998–2019) showed gastrostomy use rising from 13.7 to 22.3 per 100,000 (*p* < 0.001): PEG placements more than doubled (≈800 → 1,800/year), open gastrostomy declined (≈700 → 340/year), and laparoscopic gastrostomy increased ten-fold (≈20 → 240/year), accounting overall for 70.0% PEG, 23.3% open, and 4.9% laparoscopic of 47,800 procedures [30]. The authors attributed this rise largely to a system-wide shift toward minimally invasive techniques [30, 39]. However, international comparisons are difficult to interpret due to cultural differences and inequalities between health care systems [40].

Our data demonstrate a sustained decline in PEG use during the last two decades, raising the question of what drives this trend. While the mechanisms remain to be clarified, several lines of evidence point to contributory factors at the levels of patient selection, indication patterns, evolving nutrition pathways, and outcomes/complications—together with a cumulative “learning effect” from personal experience and national and international guidelines that has progressively steered clinicians toward more selective, goal-concordant PEG use. Since no relevant regulatory changes or changes in the reimbursement system in the German health care system occurred since the introduction of the diagnosis related groups (DRGs) in Germany in 2003, any regulatory reasons appear very unlikely. However, a special feature of the German DRG-system is a codable and reimbursed procedure for early rehabilitation during medical acute care. This procedure requires a minimum length of hospital stay of 14 days, which is the explanation for the comparably long hospital stay in geriatric acute care departments in Germany.

However, if PEG provides net benefit depends on careful selection and goals of care. In the absence of randomized trials, indications have historically been shaped by clinician judgment and experience. Over time, iterative learning and evidence based guidelines appear to have refined selection—discouraging PEG use in scenarios with low likelihood of benefit (e.g., advanced dementia) [14, 16] and prioritizing cases with potentially reversible causes of malnutrition (e.g., post-stroke dysphagia) [11]. In particular, the publication of the first German guideline on clinical nutrition in geriatrics in 2004 [41], the first European guideline on artificial enteral nutrition from 2005 [42], and the first European guideline on clinical nutrition in geriatrics from 2006 [43] may have contributed to the substantial decline in PEG insertions observed between 2004 and 2007 in our data. These guidelines emphasized careful patient selection and a restrained approach in advanced dementia. Although similar recommendations appear in later updates, the 2004–2006 documents represented the first explicit guidance cautioning against PEG in severe dementia. We cannot establish causality for Germany with the present dataset; however, clinical experience suggests that PEG was used more widely in dementia before the publication of these early guidelines.

Indications for PEG insertion may not have been static; as evidence and guideline have evolved, diagnostic profiles among PEG recipients have shifted toward conditions with clearer potential for benefit. In our cohort, stroke was the most frequent nutrition-relevant main diagnosis among PEG recipients, and dysphagia was by far the most common secondary diagnosis—aligning with prior reports that link PEG primarily to neurological dysphagia. A UK national survey similarly identified stroke and neurodegenerative conditions as leading indications; notably, 36% of hospitals reported PEG use in dementia [44]. By contrast, in our data dementia was the main diagnosis in only 0.8% of PEG recipients, and as a secondary diagnosis it declined from 40% (2006) to 15% (2024), suggesting increasingly selective use in the context of cognitive impairment and concordance with guidelines on enteral feeding in geriatric patients [13, 14].

Other comparators point the same way: in Sweden’s nationwide cohort, stroke was the largest subgroup (35), post-stroke dysphagia is common in the acute phase (reported in up to ~ 78%) [30], a German case series (*n* = 1,041) found neurogenic dysphagia (43%) and cancer (37%) as predominant indications [45], and Poland’s nationwide analysis (ICD-10–based) labeled dysphagia and malnutrition most often, likely reflecting consequences of underlying disease rather than stand-alone drivers [9]. Our results also clarify malnutrition as a label: as a main diagnosis it was uncommon (though slightly more frequent among PEG recipients), and as a secondary diagnosis it did not differ between groups—underscoring that PEG decisions are typically driven by the underlying neurologic condition and dysphagia.

Over the past two decades, routine use of oral nutritional supplements (ONS) and broader multimodal nutrition care (dietetic support, texture-modified diets, and swallowing therapy) has expanded [46]. Whereas previously PEG or parenteral nutrition were often the default options when intake was inadequate, care now typically starts with ONS and other available interventions. Even when ONS does not fully meet requirements, it can bridge transient periods of illness, allowing time for recovery and shared decision-making, and thereby reducing the need for PEG in marginal cases. This shift—toward prioritizing the oral route where feasible and reserving invasive access for clearer indications—may also have contributed to the observed decline in PEG use. Unfortunately, we are not able to prove this possible shift to more oral interventions within our data.

While PEG is generally regarded as low risk, complications are not rare in frail, multimorbid older adults, and short-term mortality remains a concern [17, 30]. In general, patients in need for PEG -insertion suffer from more severe morbidity and thus demonstrate higher mortality rates than average hospitalized patients [47, 48]. Cross-study comparisons are limited by heterogeneity in indications, age, and follow-up windows, but previous PEG studies reported 30-day mortality ranges from 3–13%: 3.3% (Taiwan) [49], 3.9% (Japan) [31], 6.5% (Brazil; Germany) [50, 51], 10.0% (Sweden) [30], and 13.0% (Canada) [52]. In our study, in-hospital mortality was 11% among PEG recipients versus 3% in non-recipients, and although mortality declined with substantial fluctuations over time (from 24.3% in 2006 to 7.4% in 2024), it remained higher in PEG recipients than in those without PEG. In our cohort, cause of death was unavailable, mortality correlated strongly with poorer cognition (lower MMSE), suggesting that underlying disease severity—rather than the procedure itself—accounts for most deaths. In the regression analysis on factors associated with mortality, PEG placement was associated with less mortality, pointing in the same direction. In light of the overall decline in in-hospital mortality after PEG insertion observed in our study, it is plausible that improved patient selection has contributed both to lower mortality and to reduced PEG utilization.

Overall, our data suggest a shift toward more selective, goal-concordant PEG use in German geriatric acute care. The present nationwide, population-based results provide a benchmark for future trials and potentially for quality control at hospital, regional, and national levels. To understand the decline of PEG use, future work should link geriatric registries with OPS-coded national counts and indication-level data to see who receives PEG, including why and how utilization changes nationally. A key open question is whether performing fewer PEGs has actually benefited patients (e.g., via lower complication rates or better goal-concordant care) or whether there are unintended consequences of not performing enteral nutrition (e.g., aspiration, malnutrition, readmissions) that offset those gains. Comparative analyses across hospitals/regions and policy contexts could help resolve this.

This study has strengths and limitations. As a nationwide, registry-based analysis, it captures a very large sample of geriatric inpatients over 18 years, enabling robust assessment of long-term trends in PEG use across Germany. Standardized ICD-10 diagnosis coding and OPS 5–431.2 procedure coding allowed consistent identification of nutritionally relevant main/secondary diagnoses and incident PEG placements during hospitalisation. The dataset also included core clinical measures (e.g., Barthel Index, MMSE), discharge destination, length of stay, and in-hospital mortality, supporting clinically meaningful comparisons.

Several Limitations have as well to be considered. First, as an observational registry study with voluntary participation, residual confounding and selection effects cannot be excluded. Second, because probable indications were inferred from ICD-10 codes, the individual clinical indication for PEG insertion could not be determined; therefore, the true “reason for PEG” cannot be established with certainty. Third, data on complications (e.g., infection, bleeding, tube dysfunction) and cause of death were not available for this analysis, limiting interpretation of safety and mechanisms. Fourth, information on feeding strategy and dose (formula type, caloric/protein targets, timing), and on refeeding-syndrome screening and management, was unavailable; thus, mortality differences could reflect underlying illness severity and/or unobserved nutritional factors. Fifth, we could not ascertain pre-existing PEG status at admission; OPS code identified newly placed PEGs during the current stay only. Sixth, the registry contains incomplete data for 2006 and 2024, and no data for 2005 due to the transition between GEMIDAS versions that year. Finally, post-discharge outcomes (e.g., 30-day mortality, readmissions, aspiration events, quality of life) were not available, precluding evaluation of longer-term benefit or harm.

## Conclusion

Over nearly two decades in Germany, PEG use in geriatric inpatient care declined substantially. This downward trend is likely multifactorial and warrants further study, but most likely reflects more restrictive patient selection, shifting indication patterns, evolving alternative nutrition pathways, and accumulating evidence on outcomes and complications, together with a cumulative “learning effect” from updated guidelines and clinical practice.

## Data Availability

The data can be accessed on request to the host of the Gemidas Pro registry, Bundesverband Geriatrie.
